# High Correlation Between Structure Development and Chemical Variation During Biofilm Formation by *Vibrio parahaemolyticus*

**DOI:** 10.3389/fmicb.2018.01881

**Published:** 2018-08-14

**Authors:** Ling Tan, Fei Zhao, Qiao Han, Aijing Zhao, Pradeep K. Malakar, Haiquan Liu, Yingjie Pan, Yong Zhao

**Affiliations:** ^1^College of Food Science and Technology, Shanghai Ocean University, Shanghai, China; ^2^Laboratory of Quality and Safety Risk Assessment for Aquatic Products on Storage and Preservation, Ministry of Agriculture, Shanghai, China; ^3^Shanghai Engineering Research Center of Aquatic-Product Processing and Preservation, Shanghai, China; ^4^Engineering Research Center of Food Thermal-Processing Technology, Shanghai Ocean University, Shanghai, China

**Keywords:** *Vibrio parahaemolyticus*, biofilm, extracellular polymeric substances (EPSs), Raman microscopy (RM), confocal laser scanning microscopy (CLSM), structure parameters, correlation

## Abstract

The complex three-dimensional structure of biofilms is supported by extracellular polymeric substances (EPSs) and additional insight on chemical variations in EPS and biofilm structure development will inform strategies for control of biofilms. *Vibrio parahaemolyticus* VPS36 biofilm development was studied using confocal laser scanning microscopy (CLSM) and Raman spectroscopy (RM). The structural parameters of the biofilm (biovolume, mean thickness, and porosity) were characterized by CLSM and the results showed that VPS36 biofilm formed dense structures after 48 h incubation. There were concurrent variations in carbohydrates and nucleic acids contents in the EPS as evidenced by RM. The Raman intensities of the chemical component in EPS, measured using Pearson’s correlation coefficient, were positively correlated with biovolume and mean thickness, and negatively correlated with porosity. The Raman intensity for carbohydrates correlated closely with mean thickness (*p*-value < 0.01) and the Raman intensity for nucleic acid correlated closely with porosity (*p*-value < 0.01). Additional evidence for these correlations were confirmed using scanning electron microscopic (SEM) and crystal violet staining.

## Introduction

Bacterial biofilms are densely populated surface-attached communities embedded in a matrix of exopolysaccharides, proteins, lipids, and extracellular DNA. These extracellular polymeric substances (EPSs) forms the backbone of a three-dimensional structure protecting the embedded bacterial cells (Flemming and Wingender, 2010; Nadell and Bassler, 2011; Flemming et al., 2016; Nadell et al., 2016). A biofilm lifecycle follows four basic stages, including reversible-irreversible attachment, microcolony formation, biofilm maturation, and dispersion (Hall-Stoodley et al., 2004; Kostakioti et al., 2013; Abdallah et al., 2014; Cappitelli et al., 2014). EPS facilitate the initial attachment of bacteria and the trapping of nutrients, and maintain the integrity of the biofilm structure under environmental fluctuations (Kostakioti et al., 2013; Han et al., 2017; Koo et al., 2017). Biofilm cells are recalcitrant and resistant to most antimicrobials and disinfectants when compared to their planktonic counterparts. Biofilm cells are extremely difficult to eradicate and these resistant properties are largely associated with biofilm structure (Elexson et al., 2014; Balsa-Canto et al., 2017). The relationship between chemical composition in EPS and structure development of biofilm is therefore important but is still not well understood.

The three-dimensional structure of biofilm is currently studied using confocal laser scanning microscopy (CLSM), scanning electron microscopy (SEM), transmission electron microscopy (TEM), and optical coherence tomography (OCT) (Ivleva et al., 2008; Efeoglu and Culha, 2013; Azeredo et al., 2017). CLSM together with image analysis software can be used to quantitatively evaluate structural characteristics of biofilms (Yun et al., 2006; Mosquera-Fernandez et al., 2016). Raman microscopy (RM) is one of the spectroscopic techniques which had been widely used in physical chemistry, materials science, biomedical science (Li et al., 2014; Butler et al., 2016; Cui et al., 2016). This promising approach provides the potential for revealing chemical composition and conformation which can be coupled to the spatial resolution of optical microscope in μm range and requires none or limited sample preparation (Ivleva et al., 2008, 2009). RM provides a distinct spectral fingerprint for all biologically relevant molecules such as proteins, nucleic acids, carbohydrates, and lipids exhibit distinct spectral features (Schuster et al., 2000; Huang et al., 2004; Rosch et al., 2005; Xu et al., 2013). RM has been applied for the analysis of the microbial communities of biofilms and for the chemical characterization of EPS (Sandt et al., 2007; Ivleva et al., 2008, 2009, 2010a). However, quantifying structural changes of biofilms to changes of the chemical composition of the EPS matrix is still lacking.

*Vibrio parahaemolyticus* is a Gram-negative halophilic bacterium that naturally thrives in marine or estuarine environments and is frequently isolated from a variety of aquatic products especially shrimps and crabs (Broberg et al., 2011). This pathogen is a leading cause of food-borne gastrointestinal illness in humans consuming raw or undercooked seafood (Su and Liu, 2007; Song et al., 2017). *V. parahaemolyticus* is also widely recognized as the common cause of food-borne illnesses in many Asian countries, including China, Japan, and Taiwan (Su and Liu, 2007; Mizan et al., 2016). This bacterium can easily form biofilm on food-contact surfaces in food processing equipment and surfaces of aquatic products (Han et al., 2017; Song et al., 2017).

We aim to quantify the progress of the *V. parahaemolyticus* biofilm lifecycle and explore the relationship between structure and chemical compositions of EPS. We hypothesize that structure is closely related to key chemical species providing the structural properties of EPS.

## Materials and Methods

### Bacterial Strains and Culture Preparation

*Vibrio parahaemolyticus* S36 strain used in this study was isolated and stored in our laboratory. The strain was maintained in Tryptic Soy Broth (TSB, Land Bridge Technology, Beijing, China) with 50% (v/v) glycerol at -80°C. *V. parahaemolyticus* S36 was isolated from shrimp samples by using specific selective media, and was identified by using species-specific gene and API system tests (BioMérieux, Marcyl’Etoile, France). The bacteria were recovered in 9 mL of TSB supplemented with 3% (w/v) NaCl and incubated overnight at 37°C with shaking at 180 rpm. Subsequently, the cell cultures were diluted with the TSB (3% NaCl) equivalent to an optical density at 600 nm (OD_600_) of about 0.4 and used in all the experiments.

### Formation and Quantification of Biofilms

In this study, biofilms formation assay were carried out as described previously (Song et al., 2017) with minor modifications. Sterile glass (GS, diameter 14 mm) was placed into 24 well polystyrene microtiter plates (Sangon Biotech Co., Ltd., Shanghai, China). Then 10 μL of the *V. parahaemolyticus* cultures (OD_600_ = 0.4) were placed in each well containing 990 μL of fresh TSB medium (3% NaCl). Each sample was tested in six replicates, the wells containing uninoculated TSB served as black control. Subsequently, the 24 well polystyrene microtiter plates were incubated at 25°C statically to form biofilms for different time (12, 24, 36, 48, 60, and 72 h) and the plates were sealed using plastic wrap to minimize evaporative loss.

Quantification of biofilms was measured by using crystal violet staining as described previously (Han et al., 2017). More specifically, after incubation, the supernatant was discarded. The glass was then washed gently three times with 1 mL of 0.1 M phosphate-buffered saline (PBS) (Sangon Biotech Co., Ltd., Shanghai, China) to remove non-fixed cells. After drying, biofilms were stained with 1 mL of 0.1% (w/v) crystal violet (Sangon Biotech Co., Ltd., Shanghai, China) for 30 min at room temperature and washed three times with 1 mL of 0.1 M PBS to remove unbound crystal violet. After drying for 30 min at room temperature, crystal violet was dissolved in 1 mL of 95% ethanol (Sinopharm Chemical Reagent Co., Ltd., Shanghai, China) for 30 min. A 200 μL suspension liquid was transferred to a 96-well microplate, and measured by BioTek Synergy 2 (Winooski, VT, United States) at 600 nm.

### Visualization of the Biofilms Using Scanning Electron Microscopy

After 12, 24, 36, 48, 60, and 72 h of static incubation at 25°C, the biofilms were fixed for 12 h at 4°C in a solution containing 4% glutaraldehyde. Then, the coupons were washed gently three times with 1 mL of sterile 0.1 M PBS. Subsequently biofilms were dehydrated in serial dilutions of 30, 50, 60, 70, 90, and 95% ethanol for 10 min each, followed by twice for 10 min rinses in 100% ethanol. The glass was then dried and finally coated with gold-palladium in an automatic sputter coater (Polaron SC7640; United Kingdom). Biofilms were visualized by using a Nova 450 scanning electron microscope (FEI, Hillsboro, OR, United States). The images were acquired for three independent replicate.

### Visualization of the Biofilms Using Confocal Laser Scanning Microscopy

After 12, 24, 36, 48, 60, and 72 h incubation, the strain VP-S36 biofilms formed on glass were washed by immersing in 1 mL of 0.1 M PBS. Then the biofilms were fixed for 30 min at 4°C in a solution containing 4% glutaraldehyde (Sangon Biotech Co., Ltd., Shanghai, China). After that, the glass was first gently rinsed three times with 1 mL of 0.1M PBS and stained with SYBR Green I (Sangon Biotech Co., Ltd., Shanghai, China) for 30 min in the dark at room temperature, after which the excess stain was removed and air dried. CLSM Images were acquired using the confocal laser scanning machine (LSM710, Carl Zeiss AG, Germany) using a 20× objective. SYBR Green I was excited using an argon laser at 488 nm and a 525 ± 25 nm band-pass filter was used to collect the emission. Then image stacking was acquired with a 1 μm thickness for each sample at six random fields of the slices. Image processing and analysis were performed using the Zen 2011 program (Carl Zeiss). The CLSM images were analyzed by the ISA-2 software (Professor Haluk Beyenal, Montana State University, United States) to determine biofilms structural parameters such as biovolume, mean thickness, porosity.

### Extraction and Analysis of EPS

To determine changes of EPS in biofilms, the EPS was extracted using the probe sonication extraction protocols (Gong et al., 2009; Han et al., 2017). Specifically, the suspended cultures were first discarded; the wells were washed with 1 mL of 0.1 M PBS to remove loose suspended cells. Subsequently, the biofilm samples were suspended in a solution of 1 mL of 0.01 M KCl by vortexing and scraping then harvested. The cells were disrupted with a sonicator (VCX 500, SONICS, Newtown, CT, United States) for four cycles of 5 s of operation and 5 s of pause at a power level of 3.5 Hz. The sonicated suspension was centrifuged for 20 min at 4,000 rcf (4°C), and the suspension was then filtered through a 0.22 μm membrane filter to ensure cell-free, EPS suspension.

### Raman Spectroscope Analysis

Extracellular polymeric substance was extracted as described in extraction and analysis of EPS. The Raman spectra of EPS were recorded with a Senterra R200-L Dispersive Raman Microscope (Bruker Optics, Ettlingen, Germany) at room temperature. A diode laser at 785 nm and 50× objective with a laser power of 3 mW was used for all Raman experiments. The Raman spectrum of each sample was calculated as the average of five measurements at different arbitrary sites on the biofilm. All Raman measurements were recorded with an integration time of 60 s in the 400–1,400 cm^-1^ range. Spectrum analysis and preprocessing of preliminary data were carried out using the Bruker OPUS software.

### Statistical Analysis

The data were expressed as the mean ± standard deviation. Correlation analysis was analyzed by the Pearson method using SPSS statistical software (version 19.0; SPSS Inc., Chicago, IL, United States). Differences at *p*-value < 0.05 were considered statistically significant. The figures were performed using Origin pro 8.0 (Origin Lab Corp., Northampton, MA, United States).

## Results

### Biofilm Formation

**FIGURE 1:**
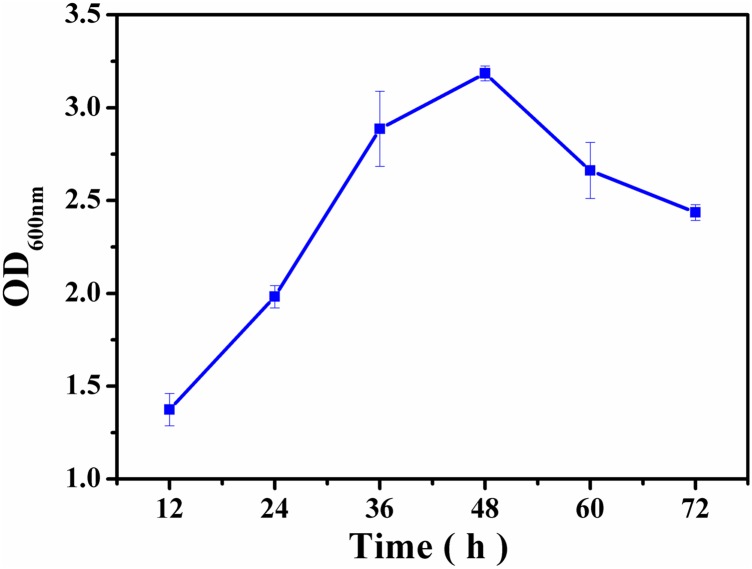
Time course of *V. parahaemolyticus* biofilm production for 12, 24, 36, 48, 60, and 72 h. Biofilm biomass (OD_600__nm_) by crystal violet staining method. The error bar represents the standard deviation of triplicate experiments.

The dynamic process of *V. parahaem*olyticus biofilm formation was followed over time using the crystal violet staining method (**Figure 1**). Stage one showed increase in biomass of the biofilm from 12 to 36 h, and confirms the bacterial attachment and growth stage of the biofilm lifecycle. Stage two could be observed from 36 to 48 h, which was the biofilm maturation stage. Biofilm diffusion stage occurred after 48 h. The crystal violet staining result showed that the biofilm lifecycle mimics a bacterial growth profile.

### Scanning Electron Microscopy Imaging

**FIGURE 2:**
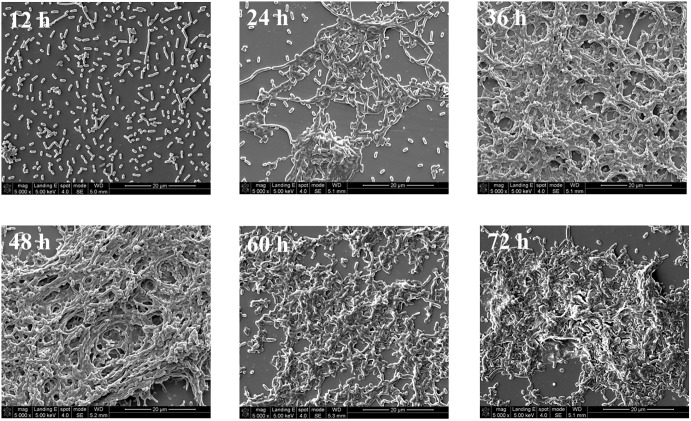
Representative photomicrographs by SEM of biofilm formed by *V. parahaemolyticus* after 12, 24, 36, 48, 60, and 72 h cultivation. Pictures were representative of three independent experiments with three replicates each.

The structural changes in the *V. parahaem*olyticus biofilm was examined by SEM (**Figure 2**) where after a 12 h incubation, the biofilm consists mostly of single cells and clustering is not common. After 24 h of incubation, the cells begin to elongate centrally and the process of clustering is more common, and there are still a scattering of individual cells. Large aggregates of cells in EPS are formed after 36 h of cultivation. A layer of mature biofilm can be seen after 48 h of cultivation where most cells adhere closely and there is a buildup of dense web-like structures within the extracellular matrix. After 60 h of cultivation, the three-dimensional framework begins to dissipate and single cell are seen detaching away from the biofilm matrix. After a 72 h cultivation period, the SEM image shows prominent cells lysis. The biofilm lifecycle measured using SEM was consistent with the observations acquired by crystal violet staining.

### Confocal Laser Scanning Microscopy Imaging

**FIGURE 3:**
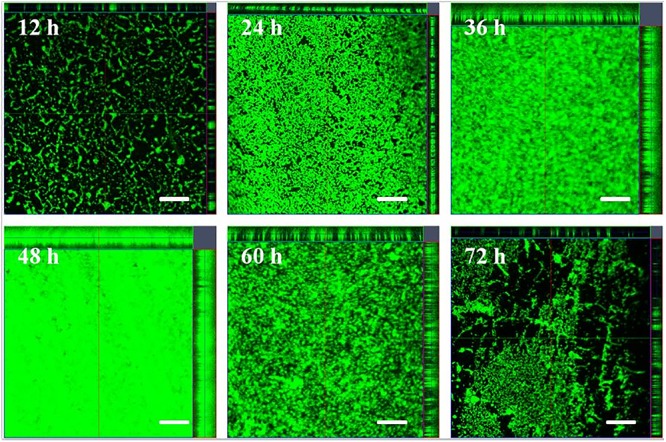
Representative CLSM images of biofilm formed by *V. parahaemolyticus* after 12, 24, 36, 48, 60 and 72 h cultivation. The scale bar represents 20 μm. The images were representative of three independent replicates.

As a parallel study, CLSM was used for examining changes to the three-dimensional structure during the development of the biofilm (**Figure 3**). At 12 h, almost all of the cells were distributed as single cells, and cell aggregation was minor. After initial attachment of bacterial cells to the surface, the biofilm started to develop and completely covered the surface at 24 h, but without large structures evident. At 36 h the cells began to cluster and formed larger structures and the biofilm had increased in thickness when comparing to the thickness at 24 h. At 48 h, the thickness of the biofilm remained similar to the thickness at 36 h, while a denser biofilm was formed. The biofilm architecture began to degrade at 60 and 72 h. This result indicated that the formation of biofilm was periodic rather than continuous.

### Characterization of Biofilm Structure

**FIGURE 4:**
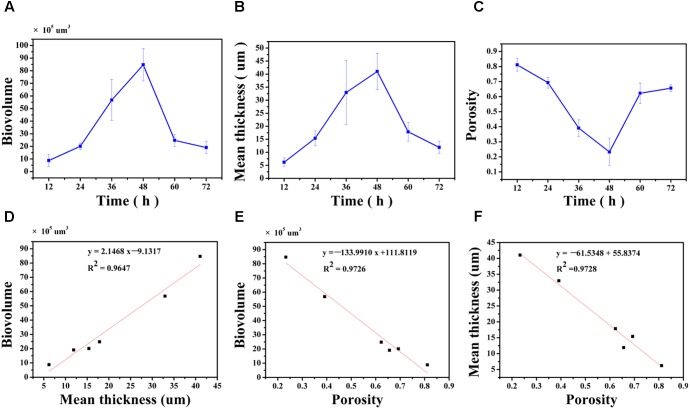
Quantification of structural parameters in biofilm formed by *V. parahaemolyticus* after 12, 24, 36, 48, 60 and 72 h cultivation **(A–C)**: **(A)** Presents the Biovolume vs. Time. **(B)** Presents the Mean thickness vs. Time. **(C)** Presents the Porosity vs. Time. Correlation between the biofilm structural parameters **(D–F)**: **(D)** Biovolume and Mean thickness, **(E)** Biovolume and Porosity, **(F)** Mean thickness and Porosity. The error bar represents the standard deviation of triplicate experiments.

The biovolume, mean thickness and porosity of the biofilm, calculated from the CLSM images, were used as proxies for quantifying morphology and structural characteristics (**Figures 4A–C**). At 12 h, the surface was covered by a biofilm with a biovolume of (8.8 ± 4.8) × 10^5^μm^3^ and mean thickness of 6.2 ± 1.7 μm. The biovolume and the mean thickness of the biofilm on the surface increased to (20.1 ± 2.5) × 10^5^μm^3^ and 15.4 ± 2.8 μm at 24 h and this increase was considered minor. After 36 h of cultivation, the biovolume and the mean thickness of the biofilm significantly increased to (56.8 ± 16.2) × 10^5^μm^3^ and 32.9 ± 12.2 μm. At 48 h, the biovolume also significantly increased to (84.7 ± 12.8) × 10^5^μm^3^, but the mean thickness only slightly increased to 41.0 ± 12.2 μm. After 60 h of cultivation, the mature biofilm was considered to have entered the biofilm degradation cycle where the biovolume and the mean thickness of the biofilm decreased to (24.8 ± 4.8) × 10^5^μm^3^ and 17.9 ± 3.6 μm when compared to the measurements at 48 h. The biofilm continue to degrade up to 72 h with resulting decrease in the biovolume and mean thickness. However, the porosity of the biofilm did not follow the same trend (**Figure 4C**). Specifically, the porosity significantly decreased from 0.81 ± 0.04 at 12 h to 0.69 ± 0.03 at 24 h. As the biofilm developed and matured, the porosity of the biofilm significantly decreased to 0.39 ± 0.06 and 0.23 ± 0.09 at 36 and 48 h, respectively, possibly indicating higher concentrations of microorganisms on the surface. Subsequently, dispersion of the biofilm resulted in an increase in porosity to 0.62 ± 0.07 and 0.66 ± 0.02 at 60 and 72 h.

The linear correlation between biovolume and mean thickness (*R*^2^= 0.9647), biovolume and porosity (*R*^2^= 0.9726), mean thickness and porosity (*R*^2^= 0.9728) were analyzed and graphically represented in **Figures 4D–F**. A high positive correlation was observed between biovolume and mean thickness (Pearson correlation coefficient 0.986) and a negative correlation was observed between biovolume and porosity (Pearson correlation coefficient -0.989) and between mean thickness and porosity (Pearson correlation coefficient -0.989) (**Table 1**).

**Table 1:** Pearson correlation matrix of biofilm structure parameters and chemical variations in EPS.

	Biovolume		Mean thickness		Porosity		Intensity_561_		Intensity_788_		Intensity_1095_	
	PCC	*p*-value	PCC	*p*-value	PCC	*p*-value	PCC	*p*-value	PCC	*p*-value	PCC	*p*-value
Biovolume	1	0^∗∗^										
Mean thickness	0.986	0.000**	1	0^∗∗^								
Porosity	-0.989	0.000**	-0.989	0.000**	1	0^∗∗^						
Intensity _561_	0.875	0.023*	0.933	0.007**	-0.907	0.013*	1	0^∗∗^				
Intensity _788_	0.887	0.019*	0.928	0.008**	-0.932	0.007**	0.977	0.001**	1	0^∗∗^		
Intensity _1095_	0.863	0.027*	0.922	0.009**	-0.897	0.015*	0.999	0.000**	0.977	0.001**	1	0^∗∗^

*Intensity_*561*,_ Intensity_*788*,_ and Intensity_*1095*_ indicate the intensity of the peak at 561 cm^-*1*^, 788 cm^-*1*^*, *and 1,095 cm^-*1*^, respectively, PCC, Pearson correlation coefficient, ^∗^p < 0.05; ^∗∗^p < 0.01.*

### EPS Analysis

**FIGURE 5:**
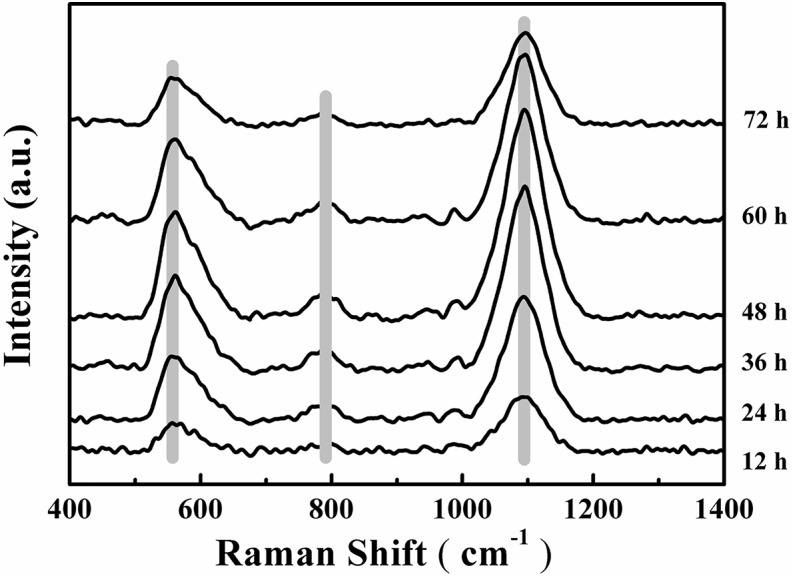
Time-dependent average Raman spectra of *V. parahaemolyticus* biofilm after 12, 24, 36, 48, 60, and 72 h cultivation. Shadow regions indicate the variation of peaks at different time points.

Changes to the Raman spectra were monitored in range of 400–1,400 cm^-1^ for quantification of the chemical composition of the biofilm and these changes are shown in **Figure 5**. The tentative peak assignments of the Raman bands are summarized in **Table 2**. Typical vibrational bands of carbohydrates, proteins and nucleic acid can be seen in the Raman spectra of biofilm. Carbohydrates accounts for two significant Raman EPS bands: 561–582 cm^-1^ and 1,090–1,095 cm^-1^ where these bands indicate a C-O-C glycosidic ring. The peak at 788 cm^-1^ indicates a O-P-O stretch of DNA which exists widely in nucleic acid (Ivleva et al., 2008, 2009; Wagner et al., 2009; Ivleva et al., 2010b; Samek et al., 2014; Han et al., 2017).

**Table 2:** Assignment of the Raman bands of EPS in biofilm matrix.

Wave number (cm^-1^)	Assignment	Macromolecular assignment	Reference
561–582	C-O -C glycosidic ring def polysaccharide; COO- wag; C-C skeletal	Carbohydrates	Ivleva et al., 2008; Ivleva et al., 2010b; Han et al., 2017
782–788	O-P -O stretch of DNA	Nucleic acids	Samek et al., 2014; Han et al., 2017
1,090–1,095	C-C str, C-O-C glycosidic link; ring br, sym	Carbohydrates	Ivleva et al., 2009, 2010b; Wagner et al., 2009; Han et al., 2017

*def, deformation vibration; str, stretching; br, breathing; sym, symmetric.*

**FIGURE 6:**
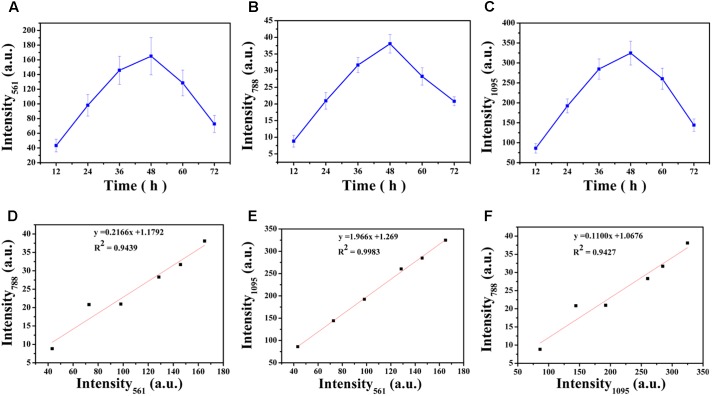
Variations in intensities of the corresponding Raman peaks over time **(A–C)**: **(A)** 561 cm^-1^, **(B)** 788 cm^-1^, **(C)** 1,095 cm^-1^. Correlation between the intensities of Raman peaks **(D–F)**: **(D)** Intensity_561_ and Intensity_788_, **(E)** Intensity_561_ and Intensity_1095_, **(F)** Intensity_1095_ and Intensity_788_. The error bar represents the standard deviation of five measurements. Intensity_561_, Intensity_788_, and Intensity_1095_ indicate the peak at 561 cm^-1^, 788 cm^-1^ and 1,095 cm^-1^, respectively.

Carbohydrates are the major component of EPS and an increase in the magnitude of Raman intensity was observed and shown in **Figures 6A,B**. Raman signals at 561 cm^-1^ and 1095 cm^-1^ increased by 2.27-fold and 2.23-fold after 24 h of cultivation and signals the accumulation of carbohydrates produced from cells in the biofilm. After 36 h of incubation, the Raman intensity increased 3.37-fold and 3.31-fold and the intensity was a maximum at 48 h. At 48 h, carbohydrate production is basically established and stable. However, after 48 h of cultivation, carbohydrate production began to decline and the Raman intensity at 60 h was similar to 36 h and the results demonstrated that 48 h was the key point for a switch of the development of maturity of biofilm. After 72 h of incubation, the carbohydrate content is less than that at 60 h and biofilm has almost degraded

The nucleic acid peak at 788 cm^-1^ was considered as a marker for this macromolecule (**Figure 6C**). Slightly weak Raman signals indicated that the content of nucleic acids increased along with the biofilm growth of *V. parahaemolyticus.* Similar results with carbohydrate production were observed with nucleic acids concentrations where after 24 h of cultivation, the Raman intensity increased by 2.37-fold. The Raman intensity increased by 3.59-fold and 4.32-fold after cultivation at 36 and 48 h and indicated that the synthesis of nucleic acid was stable at 48 h. After 48 h of cultivation, the nucleic acid concentrations began to decline.

Proteins and lipids are also major components of the EPS matrix, but Raman signals for these macromolecules were inconclusive. These results suggest that the *V. parahaemolyticus* biofilm contains little or no proteins and lipids and polysaccharides and nucleic acids were the major compounds detected by RM.

Correlation analysis revealed strong positive relationship between Intensity_561_ and Intensity_1095_ (R^2^= 0.9983; Pearson correlation coefficient 0.999), Intensity_561_ and Intensity_788_ (*R*^2^= 0.9439; Pearson correlation coefficient 0.977) and Intensity_1095_ and Intensity_788_ (*R*^2^= 0.9427; Pearson correlation coefficient 0.977). These correlations are graphically represented in **Figures 6D–F**. These relationships are positive: the higher *R*^2^, the higher degree of linear dependence, showing that the degree of dependence between polysaccharides is higher than that between polysaccharides and nucleic acids.

### Correlation Between Chemical Variations in EPS and Structures Development of *Vibrio parahaemolyticus* Biofilm

We used Pearson correlation analysis to analyze the relationship between chemical composition and biofilm structure. **Table 1** show the correlation matrix of the variables used to represent chemical composition and biofilm structure. The Raman intensity of 561 cm^-1^ (C-O-C glycosidic ring def polysaccharide) showed a significant positive correlation with biovolume (Pearson correlation coefficient 0.875, *p*-value = 0.023) and mean thickness (Pearson correlation coefficient 0.933, *p*-value = 0.007). Similar trends were detected at band 1095 cm^-1^ (C-O-C glycosidic link), which positively correlated with biovolume (Pearson correlation coefficient 0.863, *p*-value = 0.027) and mean thickness (Pearson correlation coefficient 0.922, *p*-value = 0.009). The Raman intensity of 788 cm^-1^ (O-P-O str of DNA) showed a relatively high correlation with biovolume (Pearson correlation coefficient 0.887, *p*-value = 0.019) and biofilm thickness (Pearson correlation coefficient 0.928, *p*-value = 0.008). However, the Raman intensity of 565 cm^-1^ and 1,095 cm^-1^ negatively correlated with porosity, the Pearson correlation coefficient were -0.907 and -0.897, the *p*-value were 0.013 and 0.015, respectively. Negative correlation was observed between the Raman intensity of 788 cm^-1^ and porosity (Pearson correlation coefficient -0.932, *p*-value = 0.007).

## Discussion

Biofilms on surfaces are a problem in a number of food industries as these biofilms can be a source of cross-contamination which reduces the wholesomeness of food (Srey et al., 2013; Miao et al., 2017). EPS are an integral component of biofilms and provide structural support and stability (Flemming et al., 2016; Dragos and Kovacs, 2017). In this study, we confirmed that biofilm structure is closely correlated to chemical variations in EPS.

We measured *V. parahaemolyticus* biofilm development using a combination of CLSM and RM. CLSM was the preferred technique used to characterize the structural heterogeneity of biofilm dynamically as real-time measurement can be made for measuring biofilm growth and for obtaining the three-dimensional structure of the biofilm. Furthermore, quantitative image analysis (QIA) of CSLM images provides an opportunity for characterizing the structure of the biofilm (Bridier et al., 2010). In this work, we used an ISA-2 method of QIA for quantifying structure where the biovolume and mean thickness of the biofilm increased steadily until 48 h and then began to decline due to the degradation of EPS. Our results using CLSM and image analysis showed that the *V. parahaemolyticus* biofilm follows a typical biofilm life cycle. This typical biofilm life cycle was also shown by Mukherjee et al. (2016), who developed a non-destructive microfluidic membrane flow cell in conjunction CLSM with QIA to quantify biofouling parameters such as surface coverage, biovolume, and biofilm thickness. Statistical analysis showed a positive correlation between mean thickness and biovolume (*P* < 0.01) and negative correlation between mean thickness and biovolume with porosity (*P* < 0.05) (**Figures 4D–F**). This *V. parahaemolyticus* correlations were in line with a study that evaluated biofilm structural diversity within 96 *L. monocytogenes* strains using high-throughput CLSM (Guilbaud et al., 2015). In addition, SEM was also used to examine the structure of the biofilm and results the analysis of the SEM images (**Figure 2**) confirmed the CLSM observation.

In this study, the carbohydrate and nucleic acid content in the biofilm matrix of *V. parahaemolyticus* at different stages of biofilm formation (**Figure 5**) were quantified using RM. Chao and Zhang (2012) used surface-enhanced Raman scattering (SERS) to monitor chemical variation of the Gram-negative biofilms (*E. coli* and *Pseudomonas putida*) and the Gram-positive biofilms (*Bacillus subtilis*) from the initial attachment to mature biofilm and their results showed that the lipid content in these bacterial biofilm matrix were significantly different. RM can also be used to monitor the changes in chemical composition of the biofilm when challenged with disinfectants. Han et al. (2017) observed that acidic electrolyzed water destabilizes EPS in biofilms and is an effective sanitizer. In this work, we show that biofilm structure development is closely correlated to the chemical composition in EPS and we hypothesize that manipulating this component of the biofilm will provide a basis for designing effective sanitizers.

Confocal laser scanning microscopy in combination with different chemical dyes can be used to visualize and quantify different components of the structure in biofilms, including nucleic acids and EPS (Wagner et al., 2009). Andrews et al. (2010) conducted CLSM and RM to distinguish the key macromolecules (lipids, nucleic acids, and proteins) involved in *Rhodococcus*, *Pseudomonas,* and *Sphingomonas* cell attachment to surfaces. Non-destructive detection of biofilms growth of *Pseudomonas aeruginosa* based on a Raman spectroscopic microfluidic lab-on-a-chip platform in combination with CLSM analysis was studied by Feng et al. (2015). In this study, the intensity of certain Raman bands (e.g., proteins, lipids, and carbohydrates) increased during biofilm development in the different developmental stages (i.e., early, mid, and late stages) and this increase correlated well with physiological changes measure by CLSM, namely, biofilms thickness. The spatial and temporal distribution of different EPS constituents at each stage of the *Xylella fastidiosa* biofilm life cycle were identified and characterized by Janissen et al. (2015) using different micro spectroscopic techniques, where confocal Raman intensity data revealed the presence of polysaccharides in the EPS matrix compounds. Additionally Wang et al. (2013) showed that composition and distribution of EPS is obviously associated with the incubation media during *Salmonella* biofilm development and maturation using attenuated total reflectance-Fourier transform infrared (ATR-FTIR) spectroscopy and RM.

This study shows that EPS destabilization may be the basic mechanism for the collapse of biofilm structure. Yun et al. (2006) pointed out that the spatial distribution of EPS inside the biofilm can affect filterability and control nutrient concentration fluxes. Balsa-Canto et al. (2017) and Cherifi et al. (2017) showed that under nutrient limitation, the biofilm structure of *L. monocytogenes* was enhanced but did not elaborate on the role of EPS. Therefore, future research could focus on the role of EPS in biofilm structure under nutrient limitation.

## Conclusion

In summary, we combined CLSM and RM to discover the relationship between biofilm structure and chemical composition of EPS. We conclude that there is significant correlation between chemical composition and structure development during the biofilm life cycle. Exploring this correlation deeper will give additional insight for designing more effective sanitizers for the food industry.

## Author Contributions

YZ, YP, and HL conceived and supervised the study. LT and FZ designed and performed the experiments. LT, FZ, QH and AZ analyzed the data. QH, FZ, YZ and PM revised the paper. LT wrote the paper.

## Conflict of Interest Statement

The authors declare that the research was conducted in the absence of any commercial or financial relationships that could be construed as a potential conflict of interest.
